# Disruption of *DDX53* coding sequence has limited impact on iPSC-derived human NGN2 neurons

**DOI:** 10.1186/s12920-022-01425-3

**Published:** 2023-01-12

**Authors:** Muhammad Faheem, Eric Deneault, Roumiana Alexandrova, Deivid C. Rodrigues, Giovanna Pellecchia, Carole Shum, Mehdi Zarrei, Alina Piekna, Wei Wei, Jennifer L. Howe, Bhooma Thiruvahindrapuram, Sylvia Lamoureux, P. Joel Ross, Clarrisa A. Bradley, James Ellis, Stephen W. Scherer

**Affiliations:** 1 Genetics & Genome Biology Program, Hospital for Sick Children, Toronto, ON Canada; 2 The Centre for Applied Genomics, Hospital for Sick Children, Toronto, ON Canada; 3 Centre for Oncology, Radiopharmaceuticals and Research; Biologic and Radiopharmaceutical Drugs Directorate, Health Products and Food Branch, Health Canada, Ottawa, ON Canada; 4 Developmental & Stem Cell Biology Program, Hospital for Sick Children, Toronto, ON Canada; 5 Department of Biology, University of Prince Edward Island, Charlottetown, PE Canada; 6 Neurosciences & Mental Health Program, Hospital for Sick Children, Toronto, ON Canada; 7 Department of Molecular Genetics, University of Toronto, Toronto, ON Canada; 8 McLaughlin Centre, University of Toronto, Toronto, ON Canada

**Keywords:** ASD, iPSCs, *DDX53*, *PTCHD1-AS*, CRISPR, NGN2

## Abstract

**Background:**

The X-linked *PTCHD1* locus is strongly associated with autism spectrum disorder (ASD). Males who carry chromosome microdeletions of *PTCHD1* antisense long non-coding RNA (*PTCHD1-AS*)/DEAD-box helicase 53 (*DDX53*) have ASD, or a sub-clinical form called Broader Autism Phenotype. If the deletion extends beyond *PTCHD1-AS*/*DDX53* to the next gene, *PTCHD1*, which is protein-coding, the individuals typically have ASD and intellectual disability (ID). Three male siblings with a 90 kb deletion that affects only *PTCHD1-AS* (and not including *DDX53*) have ASD. We performed a functional analysis of *DDX53* to examine its role in NGN2 neurons.

**Methods:**

We used the clustered regularly interspaced short palindromic repeats (CRISPR) gene editing strategy to knock out *DDX53* protein by inserting 3 termination codons (3TCs) into two different induced pluripotent stem cell (iPSC) lines. *DDX53* CRISPR-edited iPSCs were differentiated into cortical excitatory neurons by Neurogenin 2 (NGN-2) directed differentiation. The functional differences of *DDX53*-3TC neurons compared to isogenic control neurons with molecular and electrophysiological approaches were assessed.

**Results:**

Isogenic iPSC-derived control neurons exhibited low levels of *DDX53* transcripts. Transcriptional analysis revealed the generation of excitatory cortical neurons and DDX53 protein was not detected in iPSC-derived control neurons by western blot. Control lines and *DDX53*-3TC neurons were active in the multi-electrode array, but no overt electrophysiological phenotype in either isogenic line was observed.

**Conclusion:**

*DDX53*-3TC mutation does not alter NGN2 neuronal function in these experiments, suggesting that synaptic deficits causing ASD are unlikely in this cell type.

**Supplementary Information:**

The online version contains supplementary material available at 10.1186/s12920-022-01425-3.

## Background

Autism spectrum disorder (ASD) is a neurodevelopmental condition, which features impaired social interactions and restricted and repetitive behaviours [1] and medical complications can be involved [2]. Genetic factors can contribute an essential role in ASD development; rare (< 1% frequency) genetic variants of protein-coding genes are associated with altered function of synaptic connections, leading to ASD [3]. One locus strongly associated with ASD/ID is a broad X-linked region that includes *PTCHD1* [1], with many ASD-associated deletions upstream of that gene disrupting the neighboring long non-coding RNA *PTCHD1*-antisense (*PTCHD1-AS*) and an embedded protein coding gene DEAD-box helicase 53 (*DDX53*) [4, 5]. In some male participants with ASD, deletions upstream from *PTCHD1* eliminate the *DDX53* gene and the exons of *PTCHD1-AS* [4, 6], although a 90 kb deletion that affects only *PTCHD1-AS* (and not including *DDX53*) was found in three male siblings with ASD. *DDX53* is an intron-less 3,630 nucleotides gene that encodes a protein of 631 amino acids, containing various domains present in members of the DEAD-box helicase protein family. The latest annotation of the EAGLE system, which scores a gene’s role in ASD by studying patients having a formal ASD diagnosis classifies *PTCHD1-AS* to be strongly involved in ASD and *PTCHD1* and *DDX53* to have little formal evidence to be involved in ASD-proper [7]. Currently, *DDX53* has not been annotated in the mouse genome (either through database search or experimental testing).

Induced pluripotent stem cells (iPSCs) can give rise to any cell type through reprogramming, enabling generation of genetically matched neurons. These could be used to identify ASD-associated neuronal phenotypes [8, 9]. Human iPSC-derived neurons implicate deletions in the neurophysiology of excitatory synapses, and in ASD-associated synaptic impairment [10]. Heterozygous mutation of neurexin1(*NRXN1*) in neurons derived from human embryonic stem cells lead to reduced connectivity because of impaired neurotransmitter release [11]. Furthermore, iPSC-derived neurons from an ASD individual with disrupted *TRPC6* have reduced morphological complexity, and form fewer structural synapses [12]. Neurons haplo-insufficient for *SHANK3* have impairments in both dendrite complexity [13], and neuronal connectivity [11]. The ability of iPSCs to model developmental processes enables their use with the approach of CRISPR and CRISPR-associated protein-9 (CRISPR-Cas9) to efficiently generate knockout (KO) cell lines for disease modeling [14, 15]. Prior research using iPSC-derived neurons from participants with *PTCHD1-AS* deletions exhibit N-methyl-D-aspartate receptor hypofunction in cortical neurons and reduced miniature excitatory postsynaptic current frequency [10]. The latter phenotype was also observed in neurons where *PTCHD1-AS* was truncated by genome editing. These findings provide strong evidence that *PTCHD1-AS* deletions may be contributory for ASD, but the role of *DDX53* in neuronal function and ASD remains unknown.

The goal of this study was to evaluate the effect of introducing a disruptive variant in *DDX53* alone (while leaving *PTCHD1-AS* intact) on excitatory neuronal circuitry for a potential role in ASD causation. To avoid differences in the genetic background, we compared neurons with *DDX53* loss-of-function mutations (3TC) to isogenic controls. We used iPSCs from two individuals unaffected by ASD and edited these with CRISPR to produce two different *DDX53*-3TC cell lines. These isogenic iPSC pairs were converted to excitatory neurons by over-expression of NGN2, thereby providing a model system for functional genomic studies [9]. In isogenic control neurons, DDX53 protein levels were below the threshold for detection by western blot, and did not show a change in electrophysiological phenotype. We observed no effect of *DDX53* mutations on synaptic transmission, suggesting no overt role for *DDX53* in the autism-associated phenotypes in this excitatory neuron.

## Methods

### Reprogramming and undifferentiated iPSC culture

Two human iPSC male lines (19-2 & 50B) with different genetic backgrounds were used in this study [14]. The iPSC line 19-2 was reprogrammed from the skin fibroblasts of an unaffected father of a child with ASD who carries a de novo 16p11.2 microdeletion, associated with ASD [16]. Retroviral vectors encoding POU5F1, SOX2, KLF4, MYC, and lentiviral vectors encoding the pluripotency reporter EOS-GFP/PuroR, were used for reprogramming as described previously [17]. The iPSC line 50B was reprogrammed from an unaffected brother of a child with ASD, who was carrying a splice site mutation (c112 + 1G > C) in the gene *SET* [18]. The iPSC line 50B does not carry this mutation and was generated from CD34^+^ PBMCs at Centre for Commercialization of Regenerative Medicine (CCRM) using CytoTune™-iPSC 2.0 Sendai Reprogramming Kit [14]. These lines were CRISPR-edited and maintained in culture with their respective isogenic controls over matrigel (Corning) coated plates, changing the complete mTeSR™ (StemCellTechnologies) media every 24-h. ReLeSR™ (StemCellTechnologies) was used for cell passages in clumps, and Accutase™ (InnovativeCellTechnologies) to dissociate to single cells. Reprogramming and characterization of pluripotency, differentiation potential, and karyotype of these iPSC lines has been described [14].

### CRISPR-Cas9 design and genome editing

Depending on the type of gene editing, guide RNA (gRNA) in *DDX53* was identified by a CRISPR design tool (Benchling) (http://www.benchling.com) (Additional file 1: Table S1). Prior to nucleofection of the iPSCs with the CRISPR-Cas9 reagents, cells were treated with 10 μM Rho-associated, coiled-coil containing protein kinase (ROCK) inhibitor (RI) (Y-27632; StemCell Technologies) for 60 min at 37 °C, and dissociated in Accutase for 10–20 min. With 1.5 × 10^6^ cells for the nucleofection reaction, we incubated 1.6 μl of 10 μM gRNA with 1 μl of 20 μM Cas9-2NLS Nuclease (Synthego) in 100 μl Human Stem Cell Nucleofection Solution 1 (Lonza) for 10 min at room temperature to form Cas9 RNP complexes. Afterwards, 5 μl of 10 μM single-stranded oligodeoxynucleotide (ssODN) was added to the complexes, and nucleofected the mix into 1.5 × 10^6^ iPSCs using Nucleofector™2b (program A-023). After nucleofection, the cell solution was transferred onto the 96-well Matrigel-coated plate in mTeSR^TM^ and 10 μM RI.

### Detection of *DDX53*-3TC KO cells

**Fig. 1:**
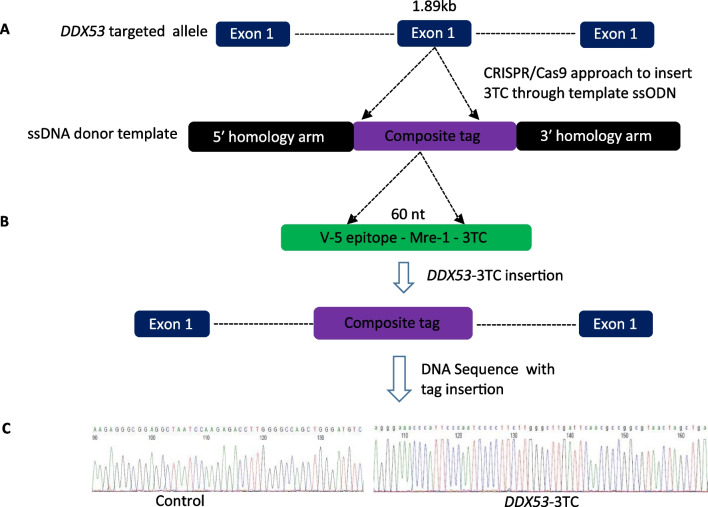
Outline of CRISPR-Cas9 experimental approach to generate *DDX53-3TC.* **A**) The *DDX53* target allele locus with CRISPR/Cas9 approach to insert 3TCs tag into *DDX53* via ssODN template. **B**) Insertion of composite tag to make a *DDX53-*3TC clone was identified by absolute quantification of tag or wild type alleles. **C**) Sequence of DNA in *DDX53-*3TC iPSC line confirms integration of composite tag within *DDX53*

**Fig. 2:**
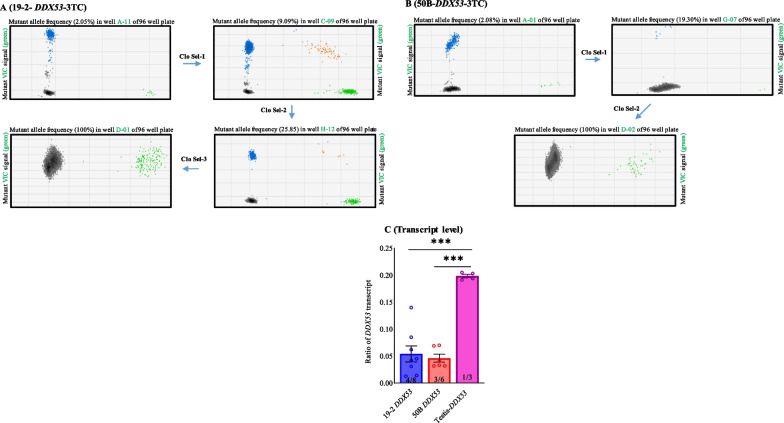
Characterization of human *DDX53-*3TC iPSCs. Blue droplets are FAM-positive droplets (WT), brown are double-positive droplets, gray are negative droplets, and green are VIC-positive droplets (mutant). (Progress of mutant allele frequency from the highest signal wells following clonal selection for 19-2-*DDX53-*3TC. **A**) and 50B-*DDX53-*3TC. **B**) Expression levels of *DDX53* gene in control lines, and testis (positive control) as a fraction of that from an internal control, *TBP* (N = 4/8 for 19-2 *DDX53*; N = 3/6 for 50B *DDX53*; N = 1/3 for testis). Numbers with each bar indicate the number of biological replicates/number of technical replicates. The *y*-axis shows the ratio of *DDX53* transcript to *TBP* transcript in different samples. **C**) ***p < 0.001, one way ANOVA with Tukey multiple comparisons test

Once iPSCs were confluent in the culture, they were treated with 10 μM RI for 60 min at 37 °C, followed by PBS washing, and treated with 30 μl/well of Accutase for 15–20 min at 37 °C. Half volume (15 ul) was transferred to each well (96-well Polymerase chain reaction (PCR) plate) of another plate containing 50 μl/well of DNA lysis buffer (10 mM Tris pH 7.5, 10 mM EDTA pH 8.0, 10 mM NaCl, 0.5% N-lauroylsarcosine), freshly added 1 mg/ml proteinase K. The other half of each well was re-seeded into a new 96-well plate, along with 250 μl/well of mTeSR™ supplemented with 10 μM RI, and maintained at 37 °C for expansion. The rest of the procedure to extract the DNA was followed, as described [14]. A DNA probe designed specific to 3TCs tag, which encompassed a Mre1 restriction site; the first half of the 3TCs of this probe was coupled with the fluorescent dye VIC, as described [14] (Additional file 1: Table S1). This design ensured high specificity of the 3TCs tag-VIC probe and secured the isolation of cells that had integrated the 3TCs at the *DDX53* gene. Conversely, a different probe was coupled with the fluorescent dye, FAM, for each wild type (wt) *DDX53* gene. Primers were designed with the primer blast tool (www.ncbi.nlm.nih.gov/tools/primer-blast/; Additional file 1: Table S1). QX-200 instrument was used (Bio-Rad, Hercules, CA) for droplet digital PCR (ddPCR) loading 20 μl ddPCR reaction mixture into the Bio-Rad DG8 droplet generator cartridge with 70 μl of droplet generation oil for each sample. These emulsions were pipetted into a separate 96-well polypropylene plate (Eppendorf), sealed with foil, and amplified in a thermal cycler (Eppendorf). Thermal cycling conditions consisted of a 10 min activation period at 95 °C, followed by 40 cycles of a two-step thermal profile of 15 s denaturation at 94 °C and 60 s at 60 °C for annealing and extension, and a final 10 min inactivation step at 98 °C. After thermal cycling, plates were transferred to a beta-prototype droplet reader to detect the frequency of modified and unmodified cells, using the absolute quantification mode. The wells with the highest proportion of *DDX53*-3TC cells were expanded into a new 96-well plate; the procedure was repeated until wells were obtained with 100% *DDX53*-3TC alleles (Fig. 2). The isolated wells with 100% *DDX53*-3TC cells were further expanded. 3TCs tag insertion (hemizygous) was confirmed by PCR on genomic DNA from KO cells, followed by sequencing (Fig. 1C).

### Potential off-target genomic sites

gRNAs were identified in the genomic region of interest by the CRISPR design tool (Benchling; http://www.benchling.com) and predicted likely off-target sites found for the gRNA within the rest of the genome. The Off-target list was used to identify the top ten off-target loci (Additional file 1: Table S2). To identify pre-selected potential off-target sites, genomic DNA was extracted from control and KO iPSCs lines using the DNeasy® kit (Qiagen), quantified and analyzed using a Qubit High Sensitivity Assay and Nanodrop OD260/280 ratio. Amplification of each primer pair (Additional file 1: Table S3) was done using wt DNA to optimize the annealing temperatures. DNA was amplified from KO cells and a control used as template to amplify regions of DNA centered on putative off-target sites, according to manufacturer's instructions (Promega). Amplified DNA was purified using the Qiaquick PCR purification kit (Qiagen), and analyzed the product using 1% agarose gel electrophoresis. Sanger sequencing of mutant lines vs wild type lines were compared to analyze predicted off-target regions.

### NGN2-lentivirus preparation

Lentivirus were produced in a HEK293T cell line by seeding 7.5 × 10^6^ cells in a T-75 flask, grown in 10% fetal bovine serum in Dulbecco’s Minimal Essential Medium (MEM) (Gibco). Next day, cells were transfected using Lipofectamine 2000 with plasmids for gag-pol (10 μg), rev (10 μg), VSV-G (5 μg), and 15 μg of the lentiviral plasmid FUW-TetO-Ng2-P2A-EGFP-T2A-puromycin or FUW-rtTA [19]. Media was changed next day, and one day later the media was harvested, cleared by filtration with a 0.45 μM filter, and lentiviral particles were pelleted in a high-speed centrifuge at 30,000 g at 4 °C for 2 h. Supernatant was discarded and 50 μl Hank's balanced salt solution Phosphate Buffered Saline added to the pellets incubating overnight at 4 °C and frozen into aliquots of 10 μl at − 80 °C the next day.

### Neuronal differentiation

Isogenic control lines and *DDX53*-3TC iPSC lines were directly converted into excitatory cortical neurons, as described [14, 20]. 5 × 10^5^ iPSCs/well were seeded in a Matrigel-coated 6-well plate in 2 ml of mTeSR supplemented with RI. The next day, media was changed with fresh mTeSR1 plus 10 μM RI, 0.8 μg/ml polybrene (Sigma), and the minimal amount of NGN2 and reverse tetracycline-controlled transactivator (rtTA) lentiviruses necessary to generate > 90% green fluorescent protein (GFP) positive cells upon doxycycline induction. The day after, virus-containing media was replaced with fresh mTeSR, and cells subcultured once they become confluent. NGN2-iPSCs (controls & *DDX53*-3TC) were treated with accutase, and seeded in a new Matrigel-coated 6-well plate at a density of 5 × 10^5^ cells/well, in 2 ml of mTeSR supplemented with 10 μM RI (day-0 of differentiation). On day-1, medium was changed in each well for 2 ml of CM1 [Dulbecco's Modified Eagle's Medium (DMEM)-F12 (Gibco), 1 × N2 (Gibco), 1 × Non-Essential Amino Acids (NEAA) (Gibco), 1 × Penicillin–Streptomycin (pen-strep) (Gibco), laminin (1 μg/ml; Sigma), brain-derived neurotrophic factor (BDNF) (10 ng/μl; Peprotech) and glial cell-derived neurotrophic factor (GDNF) (10 ng/μl; Peprotech) supplemented with fresh doxycycline hyclate (2 μg/ml; Sigma) and 10 μM RI]. On day-2, media was replaced with 2 ml of CM2 [Neurobasal of media (Gibco), 1 × B27 (Gibco), 1 × glutamax (Gibco), 1 × pen-strep, laminin (1 μg/ml), BDNF (10 ng/μl) and GDNF (10 ng/μl)] supplemented with fresh doxycycline hyclate (2 μg/ml) and puromycin (5 μg/ml for 19-2-derived cells, and 2 μg/ml for 50B-derived cells; Sigma)]. The rest of the procedure followed was as described [12].

### Droplet digital PCR for gene expression

Total RNA was extracted using Trizol reagent following manufacturer’s instructions (Thermo Fisher Scientific). cDNA was synthesized using Superscript III reverse transcriptase with oligo dT primers. For ddPCR, the reaction mix contained ddPCR master mix, *DDX53* TaqMan^®^ gene expression assay (catalogue # 4351372) (FAM), and TATA-box binding protein (TBP) TaqMan^®^ gene expression assay (VIC) (catalogue # 4448489) (ThermoFisher). 20 μl ddPCR reaction mixture was loaded into Bio-Rad droplet generator cartridge, and 70 μl of droplet generation oil for each sample. The cartridge QX200 droplet generator was transferred to a separate well of a 96-well polypropylene plate (Eppendorf), heat sealed with foil, and amplified in a thermal cycler (Eppendorf). The rest of the procedure followed as described [12]. After thermal cycling, plates were transferred to droplet reader to detect the expression of *DDX53* in controls & *DDX53*-3TC lines.

### RNA sequencing

Total RNA was extracted from each well of *DDX53*-3TC and isogenic control neurons using TRIzol (Thermo Fisher Scientific). RNA samples were processed, as described [12] with the following modifications: RNA libraries were prepared following the Illumina TruSeq Stranded total RNA library preparation protocol, 500 ng of total RNA used for rRNA depletion, and samples fragmented into 300–400 base fragments. Then, fragments were amplified under the following conditions: initial denaturation at 98 °C for 10 s, followed by 12 cycles of 98 °C for 10 s/60 °C for 30 s, and a 5 min extension at 72 °C. Data quality was assessed using FastQC v.0.11.5. Adaptors were trimmed of low quality ends with Trim Galore v.0.4.4 and screened trimmed reads for presence of ribosomal ribonucleic acid (rRNA) and mitochondrial RNA (mtRNA) sequences using FastQC Screen v.0.10.0. The RSeQC package v.2.6.2 was used to assess read distribution, positional read duplication and confirmed strandedness of alignments. Raw trimmed reads aligned to the reference genome, hg19, using STAR v.2.6.0.c. STAR alignments were processed to extract raw read counts for genes, using htseq-count v.0.6.1p2. In the filtered data set, only genes retained whose fragments per kilobase of expressed transcript per million reads of library (RPKM) values were at least 2, in at least 2 samples. Data was normalized using the Trimmed Mean of M-values (TMM) method, implemented by the calcNormFactors (y) function. The 3.1.0 ggplot2 package was used to plot a heat map, and log2-transformed the RPKM values of genes.

### Western blotting

Cells were washed in ice-cold PBS and total protein extracted in radioimmune precipitation assay (RIPA) buffer (25 mM Tris–HCl, pH7.6, 150 mM NaCl, 1% Nonidet P-40, 1% sodium deoxycholate, and 0.1% SDS) with a complete Mini protease inhibitor (Roche) [15]. 20 ug of total protein per lane was loaded on SDS-PAGE and transferred to Hybond ECL (GE HealthCare) nitrocellulose membrane. Testis lysate (Santa Cruz, Cat # SC-363781) and recombinant DDX53 (Abnova, Cat # H00168400-P01) were used as positive controls for DDX53 protein detection. Rabbit anti-DDX53 (Abcam, Cat # Ab103545, 1:1,000 dilution) and mouse anti-ACTB (Sigma, Cat # A5441) were used as primary antibodies. HRP-conjugated secondary antibodies (Invitrogen) were used and the membranes were developed with SuperSignal West Pico Chemiluminescent Substrate (Pierce). Images were acquired using a ChemiDoc MP (BioRad) and quantified using Image Lab v5.2.1 (BioRad) software. Near-Infra Red-conjugated secondary antibodies (LI-COR) were also used and membranes were scanned using LI-COR Odyssey CLx scanner according to the manufacturer’s instructions. Acquired images were analyzed using ImageStudio v5.2.5.

### Multi-electrode array (MEA)

48-well opaque-bottom MEA plates (Axion Biosystems), 16 electrodes per well, were coated with filter-sterilized 0.1% polyethyleneimine (PEI) solution in borate buffer pH 8.4 for 1 h at room temperature, washed 4 times with water, and dried overnight. The procedure followed as described [20] using Axion Biosystems with some modifications. After last reading of MEA plate at week six, wells were treated with three synaptic antagonists: Gamma-aminobutyric acid type A (GABA-A) receptor antagonist picrotoxin (PTX; Sigma) at 100 mM, α-amino-3-hydroxy-5-methyl-4-isoxazolepropionic acid (AMPA) receptor antagonist 6-cyano-7-nitroquinoxaline-2,3-dion (CNQX; Sigma) at 60 mM, and sodium ion channel antagonist tetrodotoxin (TTX; Alomone labs) at 1 mM (Fig. 5I). Plates were recorded for 10–15 min after addition of the antagonists. Culture media was replaced with CM2, and after 1 h recovery period, the final recording was taken. The weighted MFR represents the MFR per active electrode whereas burst is considered as a group of at least five spikes, each separated by an inter-spike interval (ISI) of no more than 100 ms. No non-active wells observed and therefore, no wells excluded in the analysis.

## Results

This study was prompted to assess the effect of a disruptive mutation in *DDX53* alone for a potential role in ASD causation. There is a strong genetic association of *PTCHD1-AS* variants with ASD, and a key family in which a 90 kb deletion that affects only *PTCHD1-AS* (i.e., does not involve *DDX53*) was found in three ASD-affected male siblings. To evaluate the role of *DDX53* in ASD, we knocked out the gene/protein in two different iPSC lines of two unrelated individuals who were unaffected by ASD.

### Generation of *DDX53*-3TC iPSC lines

Two iPSC lines of different genetic backgrounds were used to disrupt *DDX53* by introducing three termination codons (3TCs; Fig. 1A) to disrupt all potential reading frames of the *DDX53* gene. The insertion was targeted to nucleotide 99 of the coding sequence to truncate protein translation at amino acid 33. The inserted 3TCs were tagged with a V5 coding sequence and an Mre1 restriction site [12]. The 3TC tags were inserted through ssODN template to disrupt the *DDX53* genes of the iPSC lines (Fig. 1A and B; Additional file 1: Table S1). A single gRNA was used to direct Cas9 nuclease to make a double-strand break (DSB) in *DDX53*. Ribonucleoprotein (RNP) complex was used to deliver the Cas9 and the gRNA into iPSC lines. ddPCR was used to detect and then isolate *DDX53*-mutated cells from each line by clonal selection (Fig. 2). A single primer pair was used to amplify the region of interest and two probes to differentiate WT from mutated cells: A VIC labelled probe specific to 3TCs tag, encompassing an Mre1restriction site and the first half of 3TCs: Another FAM labelled probe was used to detect WT cells (Additional file 1: Table S1). The left homology arm of the ssODN was designed to insert a V5 tag in phase with the canonical reading frame. We previously showed that the 3TC tag could lead to reduced transcript levels of some edited genes that contain introns, and that these very small truncated proteins were never detected by the V5 antibody [12].

For cell line 19-2, a well with highest percentage of mutated cells (2%) found in the first plate, was then expanded through clonal selection into 96 wells, to increase the percentage of mutated iPSCs. 100% mutated cells was achieved after repeating the process of mutant cell isolation after four plates (Fig. 2A). For line 50B, 100% *DDX53*-3TC was obtained with three clonal selections (Fig. 2B). A complete *DDX53*-3TC clone meant a well with 100% mutated cells positive for VIC signal (green) and 0% FAM signal (blue). Isogenic controls and CRISPR-edited iPSCs were amplified by PCR. Sequencing results confirmed the integration of the 3TC tag (Fig. 1C) after 99 base pairs, stopping translation at amino acid position 33 (hemizygous).

### Characterization of off-target effects resulting from CRISPR-Cas9 editing

We devised gRNA sequences using the CRISPR designer (http://www.benchling.com) in order to minimize off-target activity. The highest-scored off-target and on-target pairs of gRNAs were selected with minimal inclusion of single nucleotide variants. These pre-selected potential off-target sites were Sanger sequenced from the PCR products. The results show that each amplicon had > 95% identity to the reference 19-2 & 50B genome, indicating no off-target effects resulting from CRISPR-Cas9 editing (Additional file 1: Table S4).

### Expression of *DDX53* in isogenic controls

Our data showed expression levels of the *DDX53* gene across control cells and testis (positive control), in relation to an internal control, TBP (Fig. 2C). We obtained ratios of the number of positive droplets of *DDX53* / number of positive droplets of TBP. As expected, amplification analysis showed that *DDX53* transcript was expressed at the low levels in 19-2 and 50B NGN2 control neurons, but was significantly higher in testis tissue. Our recent work has shown that high coverage RNAseq from MACS-enriched control iPSC-derived neurons detected *PTCHD1-AS* exon 3 in all replicates. But reads mapping to *DDX53* did not span its full exon and had different distributions in each replicate, suggesting that *DDX53* is expressed at low levels [8].

### Transcriptional characterization of isogenic controls and *DDX53*-3TC neurons

**Fig. 3:**
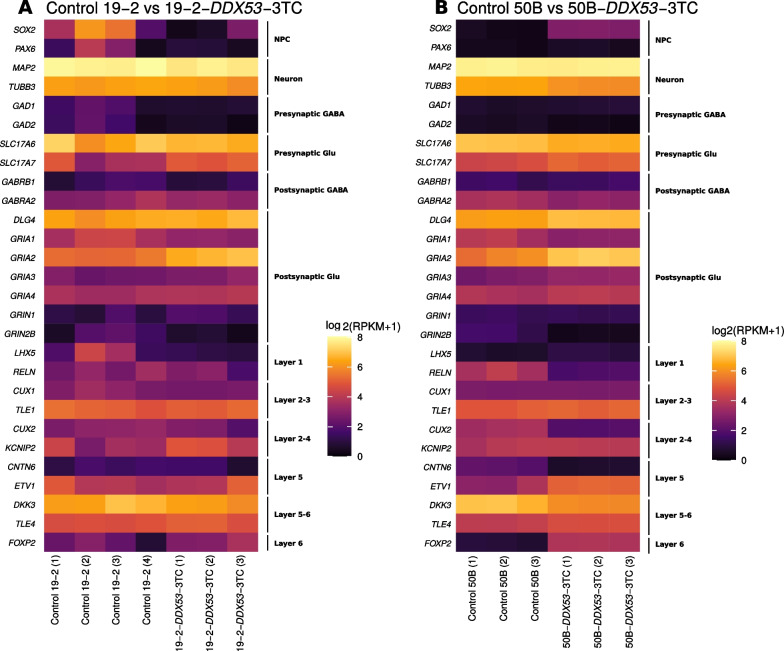
Heat map of detected RNA-Seq transcripts from *DDX53-*3TC neurons to their respective isogenic controls. Transcript levels in reads per kilo base per million (RPKM) are shown for different markers of cortical layer, neurons, neural progenitor cells (NPCs), GABAergic and glutamatergic functions. **A**) Biological replicates for isogenic controls 19-2 (n = 4) and 19-2-*DDX53*-3TC (n = 3). **B**) Biological replicates for control 50B (n = 3) and 50B-*DDX53*-3TC neurons (n = 3). (1-4) indicate the batch number

RNA sequencing (RNA-Seq) verified the glutamatergic state of iPSC-derived neurons from controls and *DDX53*-3TC neurons. Heat maps of detected RNA-Seq transcripts (reads per kilobase per million; RPKM) were generated from isogenic controls and *DDX53*-3TC neurons (Fig. 3). All cell lines show enrichment for appropriate cell differentiation markers for excitatory neuronal cell fate compared to neural progenitor cells (NPCs). All the lines showed very low levels of NPCs markers (*PAX6* and *SOX2*), and high levels of the neuronal markers (*MAP2* and *TUBB3*). GABA receptor subunit 2 (*GABRA2*) transcript level was higher compared to *GABRA1* consistent with early expression profiles of these receptors. Transcript levels for the glutamate transporter, *SLC17A6*, were high, whereas those of *SLC17A7* were lower for controls and mutant lines compared to progenitor cells. There is strong expression of α-amino-3-hydroxy-5-methyl-4-isoxazole propionic acid receptors (AMPAR) subunits (*GRIA1, GRIA2, GRIA3*, and *GRIA4*), compared to the N-methyl-D-aspartate (NMDA) NMDA receptor subunits (*GRIN1* and *GRIN2B*).

### DDX53 protein levels in controls and *DDX53*-3TC neurons

**Fig. 4:**
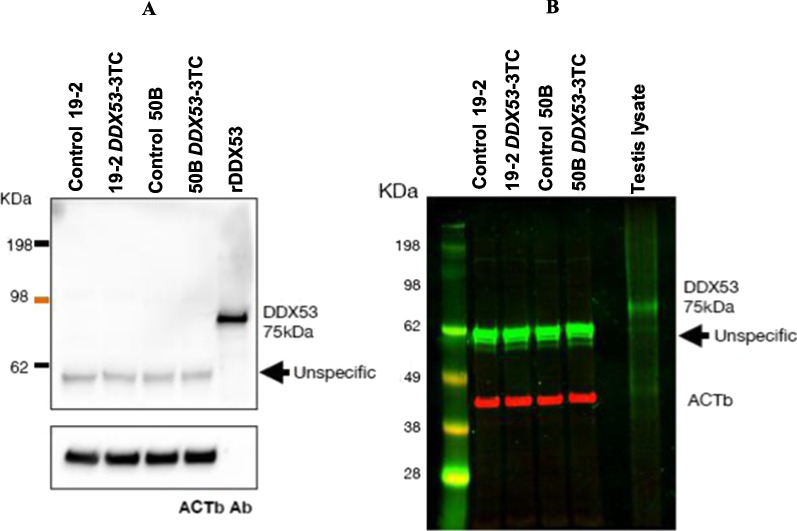
Representative western blots of DDX53 protein detection in isogenic control (Cntrl 19-2 and Cntrl 50B) and KO (19-2-*DDX53*-3TC, 50B-*DDX53*-3TC) in 4-weeks old neurons. **A**) and **B**), chemiluminescent or near-infrared detection methods, respectively. Recombinant DDX53 protein (panel **A**) and testis lysates known to express DDX53 (panel **B**) were used as positive controls. Note the appearance of an unspecific protein of approximately 60 KDa consistently detected by the anti-DDX53 antibody in both panels that does not match the molecular size of DDX53 indicated in the positive control lanes. β-actin (ACTb) was used as a loading control.

Immunoblotting was used to detect DDX53 protein in neurons derived from the isogenic pairs of control and *DDX53*-3TC mutant from each line, recombinant protein (rDDX53) (positive control; Fig. 4A), and the testis lysate (positive control; Fig. 4B). A band of the expected size (75 kDa) was observed in rDDX53, and the testis lysate (Additional file 2). There were no bands of the appropriate size for DDX53 in any of our neurons (controls & DDX53-3TC), suggesting that DDX53 protein levels are well below the threshold for detection by western blot in these cell types. Experiments showed a single unspecific band at ~ 60 kDa, expressed at similar levels in WT and *DDX53*-3TC neurons. Actin, which is highly expressed in neurons was used as a loading control.

### Characterization of the neurons to validate their excitatory nature

**Fig. 5:**
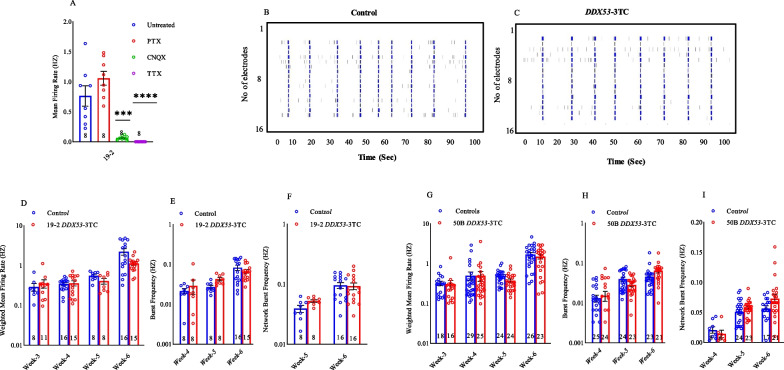
**A**) Characterization of the neurons to validate their excitatory nature: Mean firing rate of control (19-2) neurons were recorded using MEA during four consecutive readings. Recordings were done after treatment with neurotoxins. Untreated; PTX: Picrotoxin; CNQX: 6-Cyano-7-nitroquinoxaline-2, 3-dione; TTX: Tetrodotoxin (N = 8; mean ± SEM; one-way ANOVA, Sidak's multiple comparisons test). **B**) and **C**) Representative Raster plots of neuronal network activity of representative wells of control vs *DDX53*-3TC. Each row represents an individual microelectrode of the MEA device. Each black bar represents one spike and each row of black bars (spike train) represents spiking activity of a neuron, over a period of 100 s, blue bars represents the burst. Multi-electrode array analysis of *DDX53-*3TC neurons and respective isogenic controls. **D**) Weighted mean firing rate of control neurons, and *DDX53* mutant neurons (19-2-*DDX53-*3TC) from 3 to 6 weeks (N: controls for week 3 = 8; week 4 = 16; week 5 = 8; week 6 = 16. N: 19-2-*DDX53-*3TC for week 3 = 11; week 4 = 15; week 5 = 8; week 6 = 15; mean ± SEM, two-way ANOVA, Sidak's multiple comparisons test). **E**) Burst frequency of control neurons, and *DDX53* mutant neurons (19-2-*DDX53-*3TC) from 4 to 6 weeks (N: controls for week 4 = 8; week 5 = 8; week 6 = 16. N: 19-2-*DDX53-*3TC for week 4 = 8; week 5 = 8; week 6 = 15; mean ± SEM, two-way ANOVA, Sidak's multiple comparisons test). **F**) Network burst frequency of control neurons, and *DDX53* mutant neurons (19-2-*DDX53-*3TC) from 5 & 6 weeks (N: controls for week 5 = 8; week 6 = 16. N: 19-2-*DDX53-*3TC for week 5 = 8; week 6 = 16; mean ± SEM, two-way ANOVA, Sidak's multiple comparisons test). **G**) Weighted mean firing rate of control neurons, and *DDX53* mutant neurons (50B-*DDX53-*3TC) from 3–6 weeks (N: controls for week 3 = 18; week 4 = 29; week 5 = 24; week 6 = 26. N: 50B-*DDX53-*3TC for week 3 = 16; week 4 = 25; week 5 = 24; week 6 = 23; mean ± SEM, two-way ANOVA, Sidak's multiple comparisons test). **H**) Burst frequency of control neurons, and *DDX53* mutant neurons (50B-*DDX53-*3TC) from 4–6 weeks (N: controls for week 4 = 25; week 5 = 24; week 6 = 23. N: 50B-*DDX53-*3TC for week 4 = 24; week 5 = 23; week 6 = 21 mean ± SEM, two-way ANOVA, Sidak's multiple comparisons test). **I**) Network burst frequency of control neurons, and *DDX53* mutant neurons (50B-*DDX53-*3TC) from 4 to 6 weeks (N: controls for week 4 = 8; week 5 = 24; week 6 = 17. N: 50B-*DDX53-*3TC for week 4 = 6; week 5 = 23; week 6 = 21 mean ± SEM, two-way ANOVA, Sidak's multiple comparisons test)

We measured the glutamatergic/GABAergic nature of our cultured neurons to validate glutamatergic differentiation, as NGN2 ectopic expression should repress GABAergic differentiation and promote glutamatergic differentiation [21]. MFR was measured following treatment with different receptor inhibitors. There was no substantial change after the addition of the GABA receptor inhibitor, picrotoxin (PTX; Fig. 5A), indicating the absence of GABAergic transmission in our cultures. As expected, the MFR was significantly reduced in the presence of the AMPA receptor inhibitor, cyanquixaline (CNQX; Fig. 5A), which suggests that most of the cultures were composed of glutamatergic neurons, and that our Ngn-2 protocol was consistent across different cultures. All activity was abolished after the addition of the sodium channel blocker, tetrodotoxin (TTX; Fig. 5A), which effectively blocks action potentials in neurons.

### Multi-electrode array analysis of isogenic controls and *DDX53*-3TC neurons

MEA phenotyping was done to monitor the activity of the cultured neuron populations as previously described [14, 20] to characterize the *DDX53*-3TC mutant lines on the 19-2 and 50B cell line backgrounds. There are representative raster plots of neuronal network activity of representative wells of control vs *DDX53*-3TC (Fig. 5B and C). We acquired weekly recordings for all iPSC-derived neurons from weeks 3 to 6 following NGN2 induction. The highest weighted mean firing rate (wMFR), burst frequency, and network burst frequency was detected at week 6 for controls and *DDX53*-3TC neurons (Fig. 5D-I). The increase in wMFR of all neurons over the time in culture (Fig. 5D, G), indicated an increase in neuronal connectivity, consistent with culture maturation as previously observed [12]. Overall, no consistent functional differences were observed between isogenic 19-2-*DDX53*-3TC and 50B-*DDX53*-3TC lines in neuronal network activity relative to controls (Fig. 5D, G), suggesting that *DDX53* did not exert strong effects on the NGN2 neuronal phenotype. In both the controls, 19-2-*DDX53*-3TC and 50B-*DDX53*-3TC lines, burst frequency progressively increased from 4 to 6 weeks, with no difference in neuronal activity between controls or mutant lines (Fig. 5E, H). The network burst frequency was also increased from 4 to 6 weeks, but no significant difference was observed in the controls compared to 19-2-*DDX53*-3TC and 50B-*DDX53*-3TC (Fig. 5F, I).

## Discussion

We have observed a high proportion of ASD-associated deletions across the *PTCHD1-AS* locus, which may or may not impact *DDX53* [8, 22–24]. To determine whether *DDX53* has a role in the etiology of ASD in the cortex, we undertook a functional analysis of *DDX53* in NGN2 differentiated neurons to examine the synaptic phenotype. With genome editing, we inserted a triple-stop codon into the *DDX53* gene in two isogenic iPSC lines to create loss-of-function mutants. We obtained *DDX53*-3TC iPSC lines after several rounds of clonal selection, and the tag insertion was confirmed by sequencing. We examined the top 10 predicted off-target genome alterations by Sanger sequencing and found no evidence of off-target effects from genome editing.

In our transcriptional profiling for neuronal identity and maturation state, major markers for excitatory cortical fate specification appeared similar between control and *DDX53* mutant neurons. *DDX53,* has relatively limited expression in the brain [4, 6]. Our previous high-coverage RNA-Seq from magnetic-activated cell sorting (MACS) enriched control iPSC-derived mixed cortical neurons detected *PTCHD1-AS* exon 3 in all replicates, but the reads mapping to *DDX53* did not span its full exon; different distributions in each replicate suggested that *DDX53* is expressed at low levels [10]. In other public databases, such as Allen Brainspan Human Brain Atlas, *DDX53* showed very few reads under developmental transcriptome in different human brain regions. In the human protein atlas, which amalgamates data single cell RNA-seq, DDX53 is enriched in testis with some brain expression also detectable in the cerebellum. Using ddPCR we similarly observe low transcript levels of *DDX53* in control excitatory neurons relative to the high expression in testis consistent with earlier RNA expression data sets [10]. Both controls and the mutants maintained similar transcript levels of *DDX53*. DDX53 protein was not detected in our isogenic iPSC-derived control neurons when run in parallel with the recombinant protein DDX53 (rDDX53) and a testis lysate as a positive control. The neuronal lysates showed non-specific bands, with similar levels between 19-2 and 50B control NGN2 neurons. DDX53 protein was not detected in isogenic control neurons, suggesting that DDX53 protein levels are below the threshold for detection by western blot.

A study conducted by our team (Ross et al. [10]) tested the functional consequences of *PTCHD1- PTCHD1-AS* locus deletions, and iPSCs from two males with ASD were generated. Proband 1 (Prb1) has a 167 kb deletion that eliminates the promoters and first exons of *PTCHD1* and *PTCHD1-AS* [4, 16]. Proband 2 (Prb2) has a deletion that is upstream of *PTCHD1*, and eliminates the *DDX53* gene and the conserved 3rd exon of *PTCHD1-AS* [4]. The iPSC-derived neurons from these probands exhibited reduced miniature excitatory post-synaptic current (mEPSC) frequency, and NMDA receptor hypofunction [10]. Moreover, genome editing of exon 3 in iPSCs recapitulated diminished mEPSC frequency in neurons, revealing a function for *PTCHD1-AS* at excitatory synapses, and its association with ASD. The iPSCs were characterized from another Proband (Prb3) that carries an exon 3 deletion but leaves *DDX53* intact. These iPSC-derived neurons of Prb3 showed a significant decrease in mEPSC frequency with no change in amplitude, similar to that which was observed in Prb1 and Prb2 [10]. Indeed our multielectrode array results also did not show any consistent difference in *DDX53* mutant excitatory neurons compared to their isogenic controls that could explain the high penetrance of ASD at this locus. Together our results, and those of previous studies, suggest that *DDX53* has little discernable impact on excitatory synaptic transmission at this stage of development. However, *DDX53* may still be relevant and important in other cell types, such as glia, or at different stages of maturation (e.g. NPC-derived neurons) to the ASD phenotype. Extracellular recordings in the MEA system are helpful to screen for large-scale effects on synaptic transmission as it captures all neuronal activity. With whole cell patch-clamp electrophysiology, we might see some other alterations on synaptic transmission with *DDX53* mutants. Thus, future studies could employ these techniques with cell types that more robustly express DDX53 to elucidate their function in the brain.

## Conclusion

Functional analysis of *DDX53*-3TC neurons compared to isogenic control neurons did not show consistent neuronal alternations, suggesting that it has little obvious role in the glutamatergic dysregulation phenotype, and thus, suggests an impact of deletions in this region in the excitatory phenotype is due to *PTCHD1-AS*. Moreover, these iPSCs can be used to model other cell types of the brain or in other tissues that do express DDX53 protein.

## Supplementary Information

**Additional file 1.** Supplementary tables.

**Additional file 2.** Supplementary figure.

## Data Availability

RNAseq data of control samples deposited in GEO database under the accession number GSE107878. The *DDX53*-3TC KO data is available from the corresponding author on request.

## Abbreviations

ASD: Autism spectrum disorder
iPSC: Induced pluripotent stem cell
ddPCR: Droplet digital PCR
ssODN: Single-stranded oligodeoxynucleotide
RNA-Seq: RNA sequencing
rDDX53: Recombinant protein DDX53
MEA: Multi-electrode array
MFR: Mean firing rate
