# Supplementary material for: Disruption of DDX53 coding sequence has limited impact on iPSC-derived human NGN2 neurons
Source: BMC Med Genomics. 2023 Jan 12;16:5. doi: 10.1186/s12920-022-01425-3 (PMC9837974; doi:10.1186/s12920-022-01425-3)
Supplement: Supplementary file 2 — Additional file 2. Supplementary figure. [file 12920_2022_1425_MOESM2_ESM.pdf]

Supplementary information

Supplementary Table 1: The *DDX53* locus, gRNA with the PAM sequence, composite tag, ssODN template, primers, and probe sets for *DDX53* gene editing.

Supplementary Table 2: Likely off-target sites in the genome, predicted for the gRNA using Benchling.

Supplementary Table 3: Primers used to assess CRISPR/Cas9 off-target effects for *DDX53* genomic editing.

Supplementary Table 4: Off-target effects relative to the reference genome resulting from *DDX53* CRISPR editing.

Supplementary Figure 1: Full images of Western blots

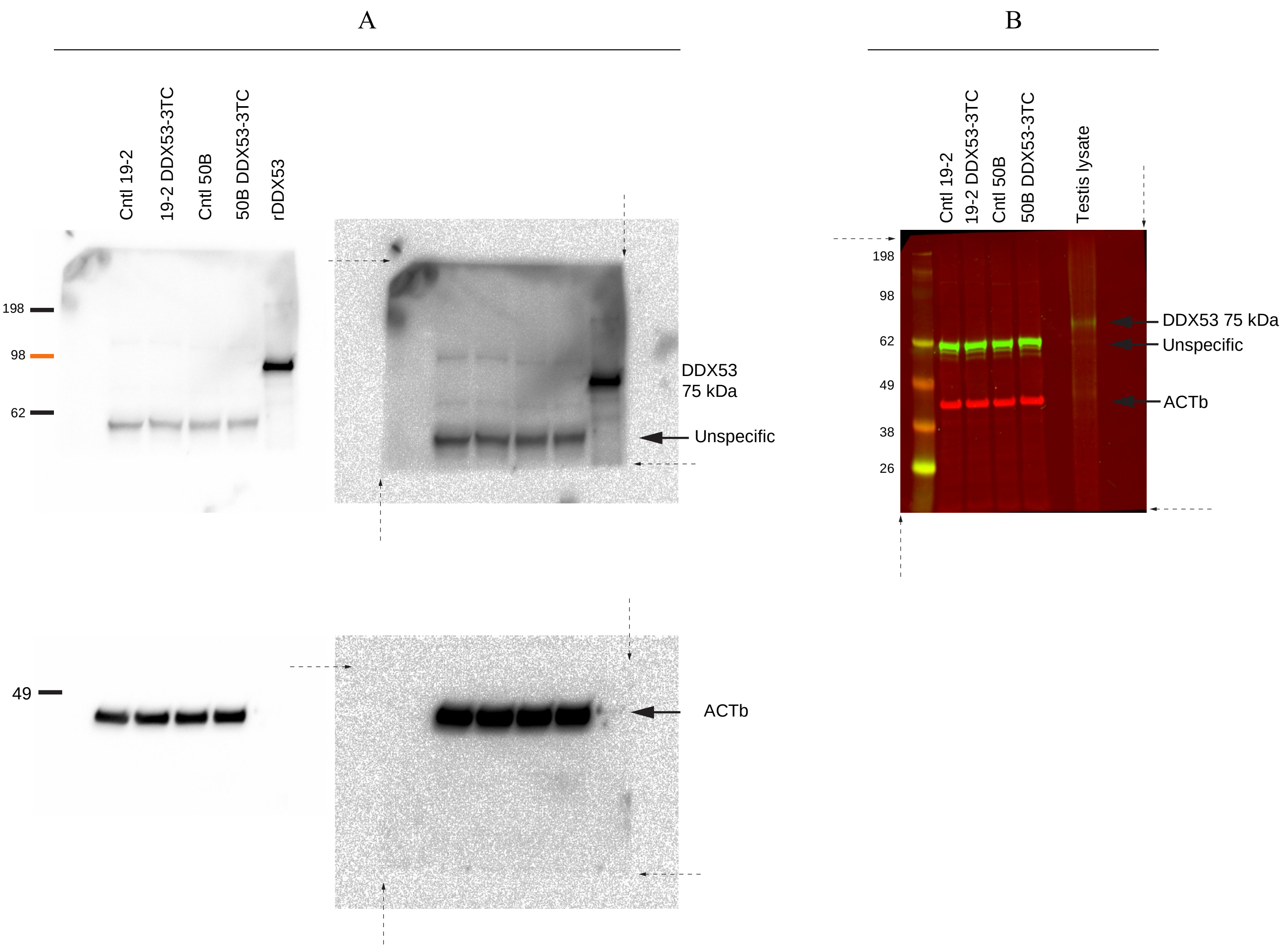
A
B
Cntl 19-2
19-2 DDX53-3TC
Cntl 50B
50B DDX53-3TC
rDDX53
198
98
62
49
DDX53
75 kDa
Unspecific
ACTb
Cntl 19-2
19-2 DDX53-3TC
Cntl 50B
50B DDX53-3TC
Testis lysate
198
98
62
49
38
26
DDX53 75 kDa
Unspecific
ACTb

Supplementary Figure 1: Western blots of DDX53 protein detection in isogenic control (Cntrl 19-2 and Cntrl 50B) and KO (19-2-DDX53-3TC, 50B-DDX53-3TC) in 4-weeks old neurons using either A) chemiluminescent with full length blots showing (left) signal optimized image or a (right) high exposure blot image to show membrane edges of recombinant DDX53 protein and B) Fluorescent detection of DDX53 with testis lysates used as positive controls. Note the appearance of an unspecific protein of approximately 60 KDa consistently detected by the anti-DDX53 antibody in both panels that does not match the molecular size of DDX53 indicated in the positive control lanes. β-actin (ACTb) was used as a loading control. Arrows indicate membrane edges.
